# Enhanced Accumulation of Colloidal Particles in Microgrooved Channels via Diffusiophoresis and Steady-State Electrolyte Flows

**DOI:** 10.1021/acs.langmuir.2c01755

**Published:** 2022-11-09

**Authors:** Naval Singh, Goran T. Vladisavljević, François Nadal, Cécile Cottin-Bizonne, Christophe Pirat, Guido Bolognesi

**Affiliations:** † Department of Chemical Engineering, Loughborough University, LoughboroughLE11 3TU, United Kingdom; ‡ Wolfson School of Mechanical, Electrical and Manufacturing Engineering, Loughborough University, LoughboroughLE11 3TU, United Kingdom; § Institut Lumière Matière, UMR5306 Université Claude Bernard Lyon 1—CNRS, Université de Lyon, Villeurbanne Cedex69622, France

## Abstract

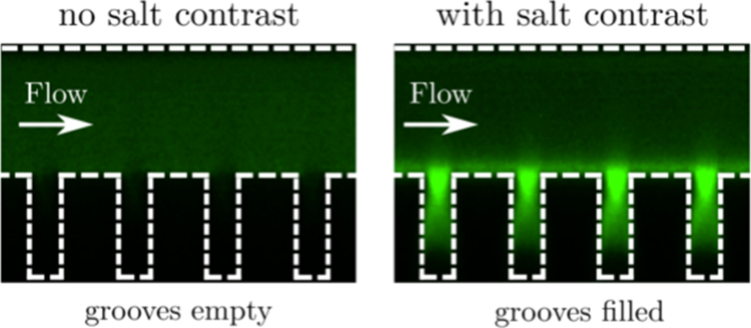

The delivery of colloidal particles in dead-end microstructures is very challenging, since these geometries do not allow net flows of particle-laden fluids; meanwhile, diffusive transport is slow and inefficient. Recently, we introduced a novel particle manipulation strategy, based on diffusiophoresis, whereby the salt concentration gradient between parallel electrolyte streams in a microgrooved channel induces the rapid (i.e., within minutes) and reversible accumulation, retention, and removal of colloidal particles in the microgrooves. In this study, we investigated the effects of salt contrast and groove depth on the accumulation process in silicon microgrooves and determined the experimental conditions that lead to a particle concentration peak of more than four times the concentration in the channel bulk. Also, we achieved an average particle concentration in the grooves of more than twice the concentration in the flowing streams and almost 2 orders of magnitude larger than the average concentration in the grooves in the absence of a salt concentration gradient. Analytical sufficient and necessary conditions for particle accumulation are also derived. Finally, we successfully tested the accumulation process in polydimethylsiloxane microgrooved channels, as they are less expensive to fabricate than silicon microgrooved substrates. The controlled and enhanced accumulation of colloidal particles in dead-end structures by solute concentration gradients has potential applications in soft matter and living systems, such as drug delivery, synthetic biology, and on-chip diagnostics.

## Introduction

The controlled transport of colloidal particles within confined environments, such as microfluidic devices, porous media, and thin films, is a key requirement in a broad range of applications, including drug discovery and delivery,^1−3^ diagnostics and therapeutics,^4−7^ enhanced oil recovery,^8,9^ filtration and antifouling technologies,^10,11^ food and cosmetic production,^12,13^ energy storage,^14,15^ environmental remediation,^16^ surface cleaning,^17^ and coatings.^18,19^ To overcome the limitations of colloid transport by mere Brownian diffusion—such as impractically low particle fluxes and lack of directionality—the dynamics of colloidal particles in microconfined geometries can be controlled through externally applied magnetic,^20,21^ electric,^22,23^ optical,^24,25^ temperature,^26^ and acoustic fields.^27,28^ Alternatively, passive methods, which do not require external actuation and rely instead on particle–fluid interactions, have been adopted to direct the particle motion in microfluidic environments. Most common passive methods include inertial^29^ and viscoelastic microfluidics^30^ and deterministic lateral displacement.^31^

In recent years, another passive transport method, called diffusiophoresis, has gained increasing attention in the colloid science and microfluidics communities.^32^ In passive diffusiophoresis,^33^ the particle motion is driven by an externally imposed solute concentration gradient, and it is governed by the interactions between the particles and the solutes. For a charged particle in an electrolyte solution, such interactions are electrostatic in nature and two distinct mechanisms contribute to the motion of the particle. First, in an electrolyte concentration gradient, a diffusion potential arises spontaneously due to diffusivity differences between the anions and cations. The electric field associated with the diffusion potential induces the electrophoretic migration of the charged particle. Second, the electrolyte concentration gradient generates a local osmotic pressure gradient and, hence, a fluid flow near the particle surface, causing the phoretic migration of the particle—a phenomenon referred to as chemiphoresis. Similarly, if a charged solid wall is exposed to an electrolyte concentration gradient, then a local electric field and an osmotic pressure gradient will be generated, thereby producing a liquid flow along the wall surface—a process called diffusioosmosis.^33^

Diffusiophoresis and diffusioosmosis have been exploited to manipulate a wide range of particle types, including solid beads,^34^ droplets,^35^ macromolecules,^36^ surfactant vesicles,^37^ liposomes,^38^ exosomes,^39^ viruses,^40^ and cells.^41^ Compared with other transport mechanisms, diffusiophoresis and diffusioosmosis offer significant advantages, especially for biological and portable point-of-need diagnostic applications, including the absence of an external energy input that requires power supply and/or bulky auxiliary equipment and ease of implementation and cost effectiveness, due to the lack of a complex and expensive setup.

The solute-driven transport of colloidal particles and liquid has been exploited for the delivery to, accumulation within, and extraction from dead-end pores^17^ and microchannels.^38,42^ These particle operations are particularly challenging since a dead-end geometry does not allow any net fluid flow. Two-step protocols, involving pore flooding and rinsing with solutions of different ionic strengths, have been proposed.^17,38^ The resulting unsteady solute concentration gradients allow to deliver just a fraction of the colloid bulk concentration of the flowing solutions within the dead-end geometry. Also, due to the transient nature of the imposed gradients, the particle manipulation capability is lost within a short period of time—typically a few tens of minutes.^43^ Longer-lasting effects and improved control over the target location can be achieved by incorporating within the dead-end channel solute-inertial beacons, which act as targets capable of maintaining long-lived solute outfluxes.^42^ Again, a two-step strategy has been adopted to first activate the target beacons and then let the particles migrate toward them. In our previous work,^44^ we introduced a new one-step strategy for the rapid (i.e., few minutes) and steady accumulation of colloidal particles within the dead-end structures (microgrooves) of a microfluidic device, consisting of a polymer microchannel glued to a silicon microgrooved substrate. In contrast with other particle focusing methods, a continuous flow setting is used to generate steady-state solute concentration gradients that allow one to retain indefinitely the control over the particle motion within the grooves. By adjusting the salt contrast between parallel flow streams, the colloidal particles can be transported into and out of the grooves multiple times without any irreversible effects, such as particle clustering and channel clogging.^45^

In this paper, we investigate the effects of groove depth and salt concentration gradient on the solute-driven particle accumulation process within the grooves. The 3D particle distribution in microchannels fitted with silicon microgrooved substrates was investigated, and the conditions to enhance the microdevice performance were determined. Furthermore, we demonstrate that the diffusiophoresis-driven accumulation of particles can be achieved also in polydimethylsiloxane (PDMS) microgrooved channels, which are easier and cheaper to fabricate compared with the silicon microgrooved substrates.

## Materials and Methods

### Materials

RTV 615 PDMS was purchased from Techsil, UK, and photoreactive Norland Optical Adhesive (NOA) 81 was purchased from Norland Products Inc., USA. The suspensions of colloidal particles were prepared with carboxylate-modified 200 nm polystyrene fluorescent colloidal spheres (Fluoresbrite YG, Polysciences, USA) and lithium chloride salt (LiCl, 99%) purchased from Acros Organics. Aqueous solutions were prepared with DI water (resistivity 18.2 MΩ cm) produced from an ultrapure Milli-Q grade purification system (Millipore, USA). The chlorinated solvent (anhydrous dichloromethane, purity ≥ 99.8%), used for recovery of the microgrooved substrate, was purchased from Sigma-Aldrich, USA.

### Fabrication of the Microfluidic Devices

**Figure 1:**
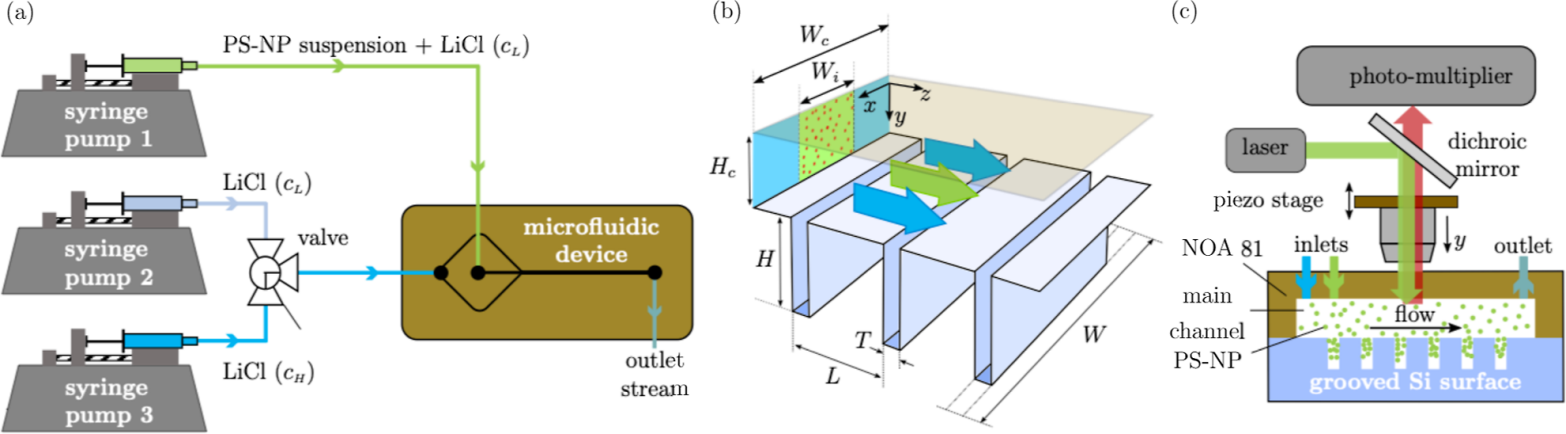
Experimental setup. (a) Syringe pumps and a flow switching valve were used to create a steady-state salt concentration gradient in a Ψ-junction device. The valve was used to switch the side streams between LiCl solutions at low (*c*_L_) and high (*c*_H_) concentrations. The central stream consisted of a suspension of carboxylated polystyrene nanoparticles (PS-NPs) at low salt concentration. (b) Geometry of the microfluidic device; the low-concentration LiCl solution seeded with PS-NPs is flowing in the central region of the channel (green shaded region), while the particle-free LiCL solution is flowing in the side regions (blue shaded regions). (c) Schematic of the confocal laser scanning microscopy rig used for particle concentration measurements within the microdevice fitted with a microgrooved surface.

The microfluidic devices (Figure 1) consisted of either an NOA 81 Ψ-junction microchannel sealed to a silicon microgrooved substrate or a PDMS Ψ-junction microchannel plasma bonded to a PDMS microgrooved substrate. The silicon substrates were purchased from FEMTO-ST Institute (Besançon, France). A total of 1250 grooves were evenly distributed on the silicon substrates over a 4 cm-long region. In all silicon substrates, the grooves had a thickness *T* = 8 μm and width *W* = 2 mm, and they were evenly spaced by a fixed distance *L* = 32 μm (Figure 1b). Substrates with a groove depth, *H*, of either 30 or 45 μm were examined in this study. The PDMS microgrooved substrates were produced via standard photo/soft-lithography techniques and characterized via scanning electron microscopy (Figure S1 in the Supporting Information). The PDMS groove thickness, width, depth, and pitch were *T* = 26 μm, *W* = 500 μm, *H* = 45 μm, and *L* = 65 μm, respectively. The NOA 81 main channels were fabricated by means of classic soft-lithography techniques. A detailed description of the fabrication process is provided elsewhere.^46,47^ In brief, a PDMS master with an imprinted Ψ-junction was fabricated via replica molding from an SU-8-coated wafer. A drop of NOA 81 was then placed on the PDMS master, and a second PDMS slab was gently pressed onto the drop for the photoreactive glue to conform to the mold shape. The NOA 81 was then partially cured through exposure to UV light. Afterward, the NOA 81 layer was peeled from the PDMS master and deposited on a microgrooved silicon substrate. Finally, the NOA 81 layer was glued to the silicon surface through a second exposure to UV light. The PDMS main channels were also fabricated via standard photo/soft-lithography techniques. SU-8 masters of the main channels and grooves, manufactured via photolithography, were used to fabricate the PDMS chips via replica molding. The PDMS channel and grooved substrate chip were then aligned and bonded together via plasma treatment. All microfluidic channels had a fixed width *W*_c_ = 400 μm, whereas the channel depth, *H*_c_, varied between 42 and 57 μm, depending on the depth of the SU-8 mold used for the fabrication process. Note that the channel width, *W*_c_, is smaller than the width of the PDMS (*W* = 500 μm) and silicon (*W* = 2 mm) grooves to facilitate the alignment between the channel and the grooved substrate. After experiments, the silicon microgrooved substrates were recovered by dissolving the NOA 81 layer in a chlorinated solvent, so that they could be further re-used for the fabrication of new microfluidic devices. Conversely, the PDMS microgrooved substrates could not be recovered. The values of the geometrical parameters of the microfluidic devices are listed in Table 1.

**Table 1:** Geometrical Parameters of the Microgrooved Channel of the Devices Used in This Study^a^

parameter	symbol	NOA/silicon devices	PDMS device (μm)
channel depth	*H*_c_	42, 52, 57 μm	45
groove depth	*H*	45 μm	45
groove thickness	*T*	8 μm	26
groove width	*W*	2 mm	500
intergroove distance	*L*	32 μm	65

a For all devices, the channel width, *W*_c_, is 400 μm and the inner (low salt) region width, *W*_i_, is 200 μm.

### Experimental Setup and Procedure

A sketch of the experimental setup used for creating a steady-state salt concentration gradient in the Ψ-junction devices is shown in Figure 1a. By means of syringe pumps (WPI, Aladdin 2-200, USA), a suspension of PS-NPs (0.025% v/v) in a low-concentration LiCl aqueous solution (*c*_L_ = 0.1 mM) was pumped in the central channel of the junction, whereas a particle-free LiCl solution at either low (*c*_L_ = 0.1 mM) or high (*c*_H_ = 10 mM) salt concentration was injected in the side channels. A flow switching valve was used to switch the side flow between the low- and high-concentration LiCl solutions. The flow rate of both the central and side streams was 12.5 μL/min. Tridimensional (3D) imaging of the particle distribution within the NOA/silicon channel and grooves was performed using a confocal laser scanning microscopy system (Leica TCS SP5, Leica Microsystems, Germany) with a 63X (NA 1.2) Leica water immersion objective (Figure 1c). The excitation and emission peaks of the fluorescent particles were 441 and 486 nm, respectively. Each vertical image sequence was recorded by scanning the distribution of the colloidal particles from the top flat wall of the microfluidic channel down to the bottom surfaces of the microgrooves (see Figure 1c). Confocal micrographs were captured as 512 × 512 pixels 16-bit TIFF images and were acquired along the vertical direction through constant incremental steps of size Δ*y* = 378 nm. The 3D colloid concentration field in the channel and grooves, *n*(*x*, *y*, *z*), was measured from the fluorescence intensity of the confocal images, *I*(*x*, *y*, *z*). A calibration curve was generated by recording the fluorescence intensity from samples of varying particle concentrations. The linearity of the relationship between *I* and *n* was confirmed within the whole range of measured intensities. The particle distribution in PDMS devices was determined via epi-fluorescence microscopy using an inverted microscope (Nikon Eclipse TE-300) fitted with an LED lamp (CoolLED pE-300), a CMOS camera (Ximea MQ013MG-ON), and a 10× (NA 0.25) Nikon objective. Again, a calibration curve, *I* versus *n*, was acquired to confirm the linear relationship between fluorescence intensity and particle concentration. Image and data analysis was performed with custom Fiji macros and Python code.

## Results and Discussion

### Particle Distribution in NOA-Silicon Microgrooved Devices

**Figure 2:**
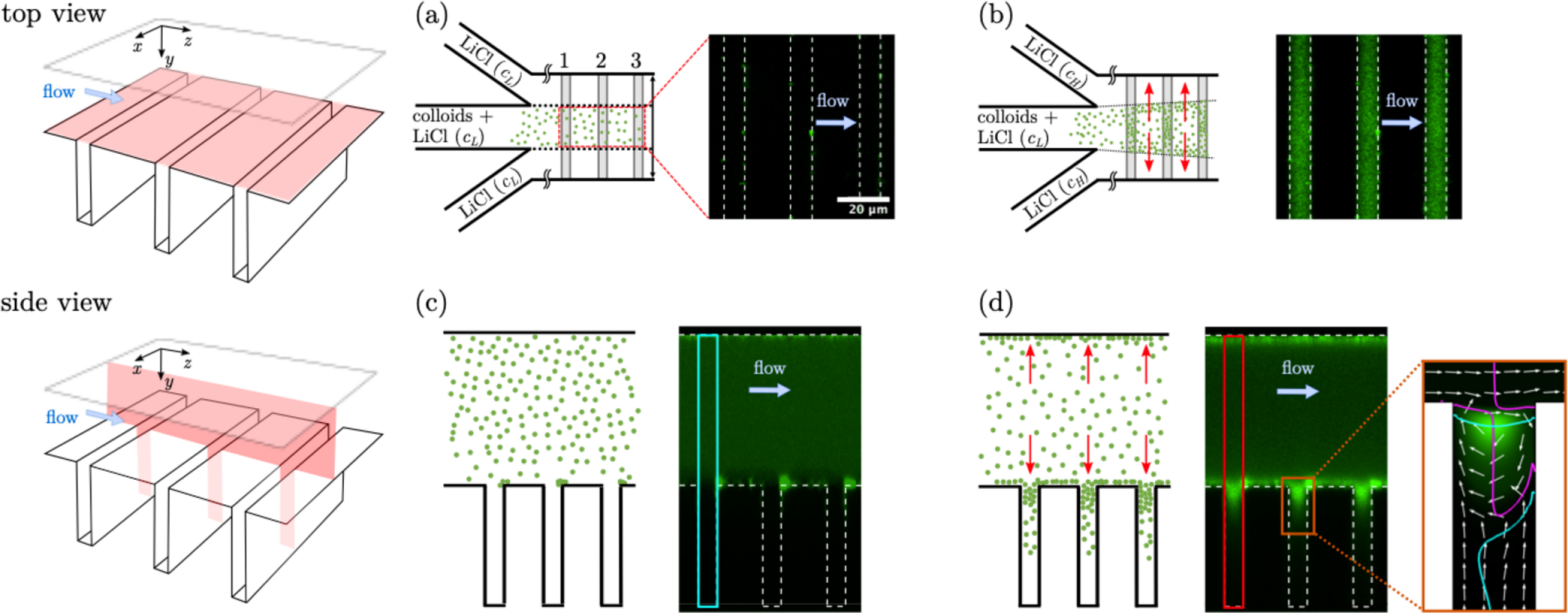
Salt-induced particle accumulation under steady-state conditions in three consecutive microgrooves located at 4 mm from the Ψ-junction of an NOA/silicon device. The 3D schematics show the orientation of the viewing planes for the top and side views. The 2D schematics and confocal images show the particle distribution without salt contrast (a,c) and with salt contrast (b,d). Red arrows show the direction of the salt concentration gradient. White dashed lines indicate channel boundaries and groove edges. In top views, fluorescence intensities are averaged over the groove depth, *H*. In side views, intensities are averaged over the range *x*/*W*_c_ ∈ [−0.2, 0.2]. The same color scale applies to all micrographs. Channel depth, *H*_c_ = 57 μm; groove depth, *H* = 45 μm; and salt concentration in solutions, *c*_L_ = 0.1 mM and *c*_H_ = 10 mM. The inset in panel (d) shows the particle concentration, predicted by numerical simulations,^44^ in a single groove together with the streamlines (white arrows) of the particle velocity ***u***_p_. The blue (magenta) solid lines show the points where the *z* component (*y* component) of ***u***_p_ vanishes.

The confocal cross-sectional images in Figure 2 show the typical redistribution of particles within a Ψ-junction microgrooved device in the presence or absence of a salt concentration gradient. In this experiment, an NOA-silicon device with a channel depth *H*_c_ = 57 μm and a groove depth *H* = 45 μm was used. The low and high salt concentrations were *c*_L_ = 0.1 mM and *c*_H_ = 10 mM, respectively. When the inner and outer flows, injected in the Ψ-junction, have the same salt concentration, *c*_L_, the particles remain homogeneously distributed within the channel without entering the grooves (Figure 2a,c). This behavior is likely due to steric effects and the electrokinetic lift of colloids near the negatively charged silicon surface.^48^ Switching the side flow stream to the solution at high salt concentration, *c*_H_, triggers the redistribution of colloids within the device due to diffusiophoresis motion (Figure 2c,d). In this configuration, the colloids migrate toward regions of higher salt concentration, *c*, with a diffusiophoresis velocity, ***u***_DP_ = Γ_DP_**∇** ln *c*, where the diffusiophoresis coefficient, Γ_DP_, is positive under the examined experimental conditions. The red arrows in the schematics of Figure 2b,d show the direction of the main components of the salt concentration gradient, namely, one along the transverse (*x*) axis and one along the depth (*y*) axis. The latter component of the salt concentration gradient is originated by the Poiseuille-like velocity profile in the rectangular channel.^44^ Consequently, the diffusiophoresis-driven particles not only spread along the transverse direction but also migrate toward the channel’s flat wall at the top and the grooved surface at the bottom, where they accumulate just below the entrance of the grooves (Figure 2d). Note that small particle aggregates form at the right top edge of the grooves, as shown by the bright spots above the groove entrance in Figure 2c (no salt contrast) and Figure 2d (with salt contrast). Silicon surface fouling at these specific locations might be caused by the particle velocity field (inset Figure 2d), which advects the particles toward the groove edges, thus facilitating particle–particle and particle–surface adhesion.

In our previous study,^44^ we conducted a finite element investigation of the microfluidic system to understand the physical mechanism driving the particle accumulation within the grooves. In brief, as the particles travel by diffusiophoresis down the groove along the concentration gradient, they are captured by the closed flow streamlines in the recirculation region at the entrance of the groove (inset in Figure 2d). A diffusioosmosis counterflow, directed upward (i.e., from the groove toward the channel), prevents the accumulation of the particles at the dead end of the groove; therefore, particles are mainly localized near the groove entrance. The competition between the particle diffusiophoresis velocity, the diffusioosmosis flow, and the particle Brownian motion determines the intensity of the accumulation peak. Interestingly, the in-plane (*y* and *z*) components of the particle velocity vanish at the particle concentration peak, but the out-of-plane (*x*) component is non-zero at the focusing point and everywhere else in the groove. Therefore, the particles are continuously advected in and out of the focusing region.^44^

Previous studies^39,45,49^ showed how colloidal particles can get trapped at the point where the hydrodynamic velocity, ***u***, and diffusiophoresis velocity, ***u***_DP_, balance each other—that is where the total particle velocity ***u***_p_ = ***u*** + ***u***_DP_ is zero—and the divergence of the particle velocity is negative. However, the set of conditions, ***u***_p_ = 0 and **∇**·***u***_p_ < 0, may not be sufficient nor necessary for particle accumulation. Indeed, in our system, particle focusing occurs at a point of non-zero particle velocity (***u***_p_ ≠ 0). Consequently, it is necessary to determine a new set of conditions on particle velocities for having an accumulation peak. If we consider the case of an incompressible flow in one-dimensional domain, it can be demonstrated (see Appendix A) that the double condition
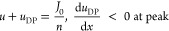
1is both necessary and sufficient for having a local maximum in the particle concentration. In eq 1, *J*_0_ denotes the particle flux at the domain’s boundaries—note that the particle flux is also constant through the whole domain. It follows that only in the case of zero particle flux at the domain’s boundaries (*J*_0_ = 0), eq 1 can be rewritten as
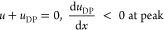
2which matches the assumption on particle focusing conditions adopted in previous studies. However, in the case of a non-zero total flux *J*_0_, the particle velocity at the peak is non-zero and the local maximum in particle density is located where the condition in eq 1 is satisfied.

### Effects of Salt Contrast and Groove Depth on Trapping Performance in NOA-Silicon Microgrooved Devices

**Figure 3:**
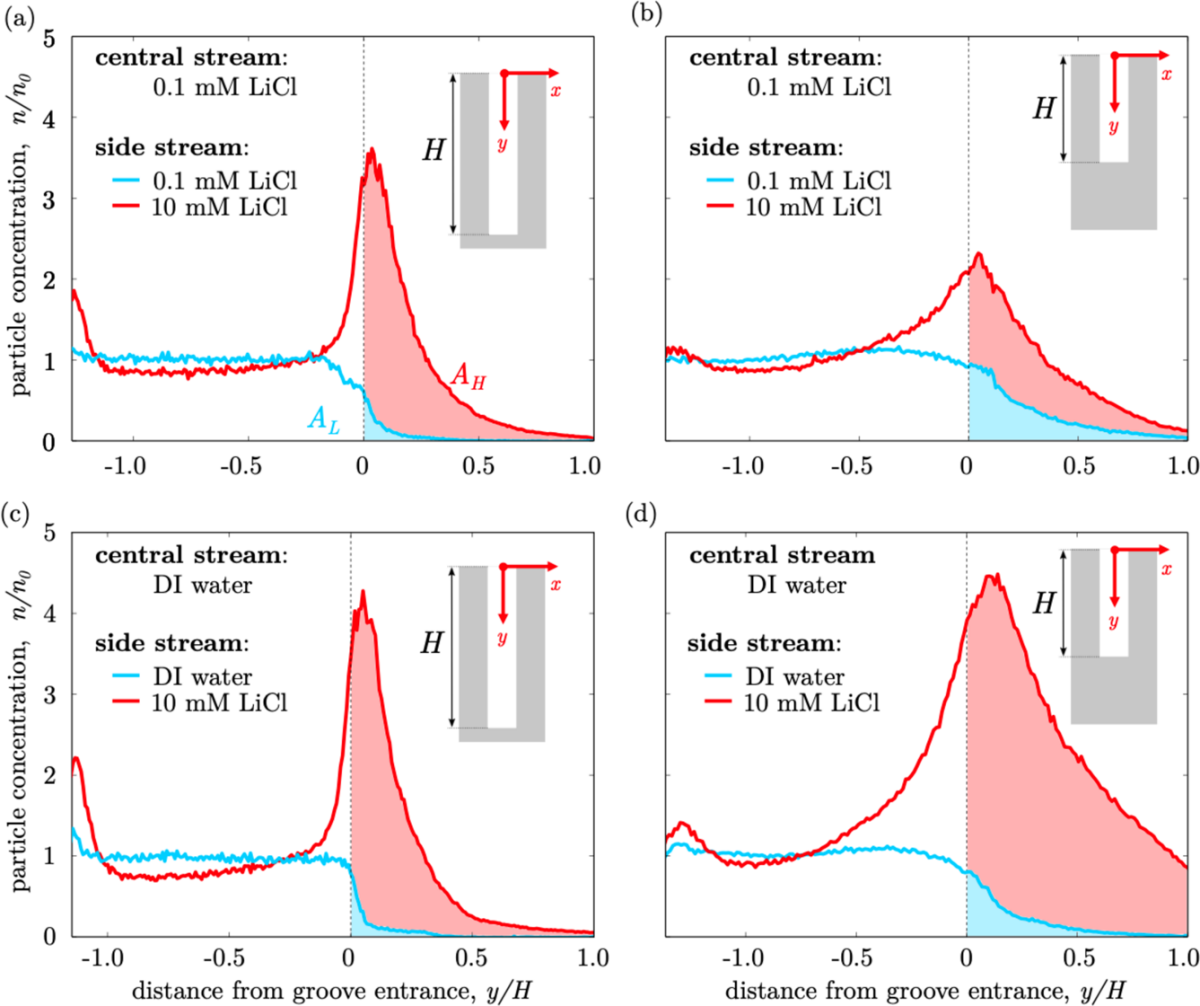
Normalized steady-state particle concentration profiles along the depthwise direction (*y*) for devices fitted with 45 μm (a,c) and 30 μm (b,d) deep grooves. The groove entrance is located at *y* = 0. The blue curves are the profiles without salt concentration gradients, and the red curves are the profiles with a lower salt contrast (a,b) and a higher salt contrast (c,d). In panel (a), *A*_L_ and *A*_H_ are the integral of the profiles within the groove (*y* ≥ 0) without (blue shaded region) and with (red shaded region) the salt concentration gradient, respectively. Channel depths: (a) 57 μm, (c) 52 μm, and (b,d) 42 μm.

**Figure 4:**
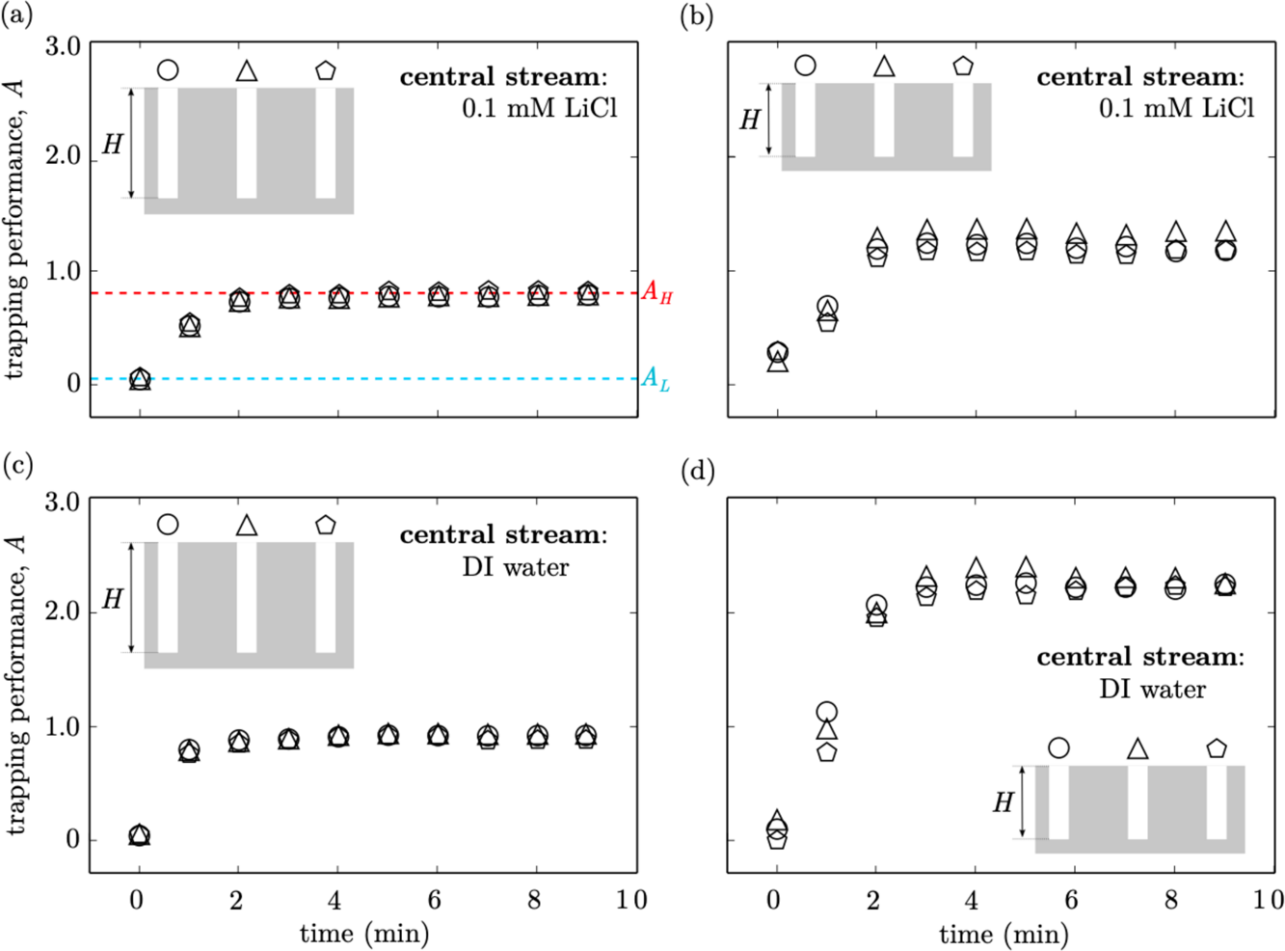
Time evolution of trapping performance of three neighboring grooves (identified by different symbols) under varying groove depths and low salt concentrations *c*_L_. (a) *H* = 45 μm, central stream: *c*_L_ = 0.1 mM, (b) *H* = 30 μm, central stream *c*_L_ = 0.1 mM, (c) *H* = 45 μm, central stream *c*_L_ = 0, and (d) *H* = 30 μm, central steam *c*_L_ = 0. At time *t* = 0, the side stream is *c*_L_ = 0.1 mM for (a,b) and *c*_L_ = 0 for (c,d). At any time *t* > 0, the side stream is *c*_H_ = 10 mM for all cases.

A set of experiments were conducted to investigate particle accumulation within the grooves under varying experimental conditions and to identify those conditions resulting in the best trapping performance. More specifically, we examined how the particle distribution and the groove trapping performance are affected by the salt concentration gradient intensity and the groove depth in NOA-silicon devices. To quantify the trapping performance of the grooves, let us consider the steady-state particle concentration profiles along the channel depth direction (*y* axis) for the NOA-silicon device of Figure 2, having a groove depth *H* = 45 μm and exposed to a salt concentration contrast of *c*_L_ = 0.1 mM and *c*_H_ = 10 mM. These particle concentration profiles are shown in Figure 3a, in case of no salt contrast (blue curve) and after imposing the salt concentration gradient by switching the flow valve (red curve). They are calculated by averaging the particle concentration field, *n*(*x*, *y*, *z*), over the *x* range of the confocal images (ca. *x*/*W*_c_ ∈ [ −0.2, 0.2]) and over the *z* range corresponding to the groove thickness *T*—as also highlighted by the solid rectangles in Figure 2c,d. The colloid concentration profiles are then normalized with respect to the original bulk colloid concentration, *n*_0_, of the colloidal solution initially injected into the device. Without salt contrast, the particle concentration profile is constant (*n* ≃ *n*_0_) throughout the channel, apart from the colloid-depleted region near the entrance of the groove (*y* → 0). On the other hand, the salt concentration gradient induces the migration of particles from the channel bulk toward the top flat wall (*y* → −*H_c_*) and the groove (*y* → 0). As a result, two accumulation peaks appear at these locations. The trapping performance of the grooves can be quantified via a trapping performance parameter, *A*, defined as the area below the profiles for *y* > 0 (shaded regions) in Figure 3a. Note that, by definition, the trapping performance parameter is equal to the average particle concentration within the groove, normalized with respect to *n*_0_. The parameters *A*_L_ (the blue region area) and *A*_H_ (the red region area) correspond to the trapping performance in the absence and presence of the solute concentration gradient, respectively. Figure 4a shows the time evolution of the trapping performance for three consecutive grooves when the salt concentration gradient is applied by switching the flow valve at the time *t* = 0. It is worth noting that by swapping the outer flow stream alternatively between low (*c*_L_) and high (*c*_H_) salt concentration solutions, the trapping performance varies cyclically between the low (*A*_L_) and high (*A*_H_) values (see Figure S2 in the Supporting Information). This implies that the particle focusing phenomenon is fully reversible, and the trapping and extraction of the colloidal particles in the microgrooves can be controlled by simply altering the solute concentration gradient via the flow valve.

A second NOA-silicon Ψ-junction device with shallower grooves (i.e., groove depth *H* = 30 μm) was fabricated to investigate the effect of groove depth. The other dimensions of the grooves were kept the same as in the first device (i.e., *T* = 8 μm and *L* = 32 μm). The performance of both devices (i.e., *H* = 30 μm and *H* = 45 μm) was examined under varying salt conditions, namely, the high salt concentration *c*_H_ = 10 mM was kept constant, whereas the low salt concentration, *c*_L_, was either 0.1 mM (i.e., lower salt contrast) or 0 (i.e., higher salt contrast). The normalized steady-state particle concentration profiles for the two devices, without a salt concentration gradient (blue curves) and under lower and higher salt contrast conditions (red curves), are shown in Figure 3. All profiles refer to grooves located at 4 mm from the junction. As expected, in the absence of a salt concentration gradient, particles are homogeneously distributed in the channel, and they hardly penetrate into the grooves. Under steady-state salt concentration gradients, all profiles share similar features, including the two accumulation peaks—one near the top flat wall (*y* < 0) and one below the entrance of the groove (*y* > 0)—and a slight depletion of particles (*n*/*n*_0_ < 1) in the bulk of the channel. Such a depletion is the consequence of the diffusiophoresis-driven particle spreading along the transverse (*x*) direction, an observation consistent with previous studies on Ψ-junction devices with similar flow configurations.^34^ The key parameters characterizing the particle concentration peaks within the grooves are reported in Table 2 for all examined cases.

**Table 2:** Properties of the Particle Concentration Peaks Within the Grooves for Varying Groove Depths, *H*, and Low Salt Concentrations, *c*_L_^a^

	*H* = 45 μm		*H* = 30 μm	
	*c*_L_ = 0	*c*_L_ = 0.1 mM	*c*_L_ = 0	*c*_L_ = 0.1 mM
*n*_max_/*n*_0_	4.00 ± 0.16	3.48 ± 0.06	4.65 ± 0.03	2.40 ± 0.15
*y*_peak_/*H*	0.052 ± 0.004	0.051 ± 0.003	0.125 ± 0.002	0.050 ± 0.03
*A*_H_	0.93 ± 0.01	0.79 ± 0.03	2.30 ± 0.02	1.25 ± 0.07
*A*_H_/*A*_L_	42	36	75	51

a The corresponding concentration profiles are shown in Figure 3. Peaks are characterized in terms of peak intensity *n*_max_, peak position along channel depth direction *y*_peak_, and trapping performance parameter without (*A*_L_) and with (*A*_H_) a salt contrast.

For a given device geometry, the intensities of the particle peaks near the flat walls and within the grooves, *n*_max_, increase with the salt contrast (Figure 3a,c), due to the increased diffusiophoresis velocity, ***u***_DP_, of the particles. Conversely, the location of the peaks within the grooves, *y*_peak_, is rather insensitive to the salt concentration gradient and groove depth. This observation is in agreement with the findings from our previous numerical study^44^ for which the particle peak is located at the center of the flow recirculation region within the groove and is independent of the diffusioosmosis flow. Also, due to the large aspect ratio of the grooves (*H*/*T* > 3.5), the groove depth has little effect on the recirculation flow at the entrance of the groove. As discussed in the previous section, the diffusioosmosis flow prevents the accumulation of particles at the dead end of the groove, and the particle trapping phenomenon is restricted to the flow recirculation region only. Therefore, for 45 μm-deep grooves with a relatively high aspect ratio (*H*/*T* = 5.6), the volume of the groove near the dead end (*y*/*H* → 1) does not contribute to the trapping effect, as shown by Figure 3a,c. Thus, it is expected that shallower grooves with a lower aspect ratio (*H*/*T* = 3.8 for the 30 μm deep groove) result in a more efficient use of the groove volume, leading to higher trapping performances, *A*. This prediction is confirmed by Figure 4, which shows the time evolution of the trapping performances of three consecutive grooves, at 4 mm from the junction, for both devices under different salt conditions. For a given groove depth, higher salt concentration gradients result in a higher trapping performance, and for a given salt contrast, shallower grooves lead to increased trapping performances. It can be concluded that, among the tested conditions, the highest trapping performance parameter *A* (i.e., the normalized average particle concentration within the groove) is achieved for a 30 μm-deep groove under a salt contrast of *c*_L_ = 0 and *c*_H_ = 10 mM. Under these experimental conditions, a ca. 75-fold increase in the groove’s average particle concentration is achieved within a few minutes after the imposition of the external steady-state salt concentration gradient. The resulting trapping performance parameter *A* is 2.3 ± 0.02, which means that the average particle concentration within the grooves is 2.3 times higher than the particle concentration, *n*_0_, of the original colloidal solution injected into the device. Importantly, these experimental conditions also lead to the highest concentration peak, with particles at the focusing point being 4.65 times more concentrated than in the original colloidal solution (Figure 3d). Note that the maximum particle concentration remains well below the packing limit, thereby avoiding irreversible effects such as particle clustering and device clogging.

From the time evolutions of the trapping performance in Figure 4, it can be observed that steady-state particle distributions are achieved typically within 2 or 3 min after switching the outer stream valve. As an example, for *H* = 30 μm and *c*_L_ = 0 (Figure 4d), the particle accumulation rate is *n*_0_*A*/τ_trans_ ≃ 0.2% min^–1^, with *n*_0_ = 0.025%(v/v), *A* = 2.3, and τ_trans_ = 3 min the duration of the transient regime. To understand the mechanisms determining the particle accumulation rate, we estimated the hydrodynamic advection and diffusiophoresis transport rate from the microchannel to the grooves (see the Supporting Information). Assuming a steady salt concentration field, the characteristic time for particle migration from the channel to the groove is about 7 s, which is much smaller than the transient times observed in the experiments. On the other hand, the time required by the outer stream with higher salt concentration to travel from the switching valve to the device is about 1 min, namely, the same order of the transient times observed experimentally. We conclude that the rate-determining step of the particle accumulation process is the advective transport of the outer stream from the valve to the device rather than the advective and diffusiophoresis transport of particles from the microchannel to the grooves.

Finally, it is worth noting that colloidal particles with a very low (Γ_DP_ ≃ 0) or even negative (Γ_DP_ < 0) diffusiophoresis coefficient should not accumulate within the grooves. Consequently, the trapping performance for these particles should be similar to the one of negatively charged particles in the absence of the salt concentration gradient, namely, *A*_L_ (see also Figure 4a). This suggests a potential application of our microdevice for the separation of colloids based on surface charge or size, which both affect the diffusiophoresis coefficient. Theoretically, a feed stream of a 1:1 (v/v) mixture of type 1 (negatively charged) and type 2 (neutral or positively charged) colloids could lead to a *A*_H_:*A*_L_ mixture of type 1 and 2 colloids within the grooves—*A*_L_ and *A*_H_ being the trapping performance parameters for the negatively charged (type 1) colloids without and with salt contrast, respectively. The corresponding separation efficiency could be defined as , and for the examined experimental conditions (Table 2), one should expect separation efficiencies within the range from 97 to 99%. These predictions are very promising, but future investigations are necessary to quantify experimentally the performance of these devices for separation applications.

### Particle Accumulation in PDMS Microgrooved Devices

**Figure 5:**
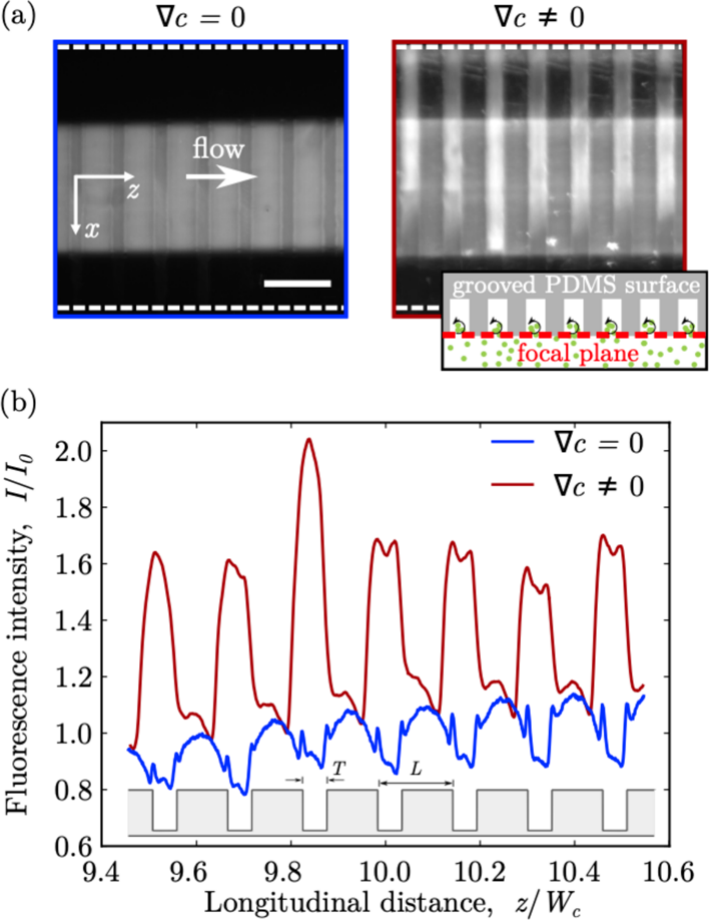
Diffusiophoresis-driven accumulation of colloidal particles in a PDMS microgrooved channel. (a) Epi-fluorescence micrographs of the main channel at ca. 4 mm (*z*/*w* = 10) downstream of the junction, without and with a steady salt concentration gradient, **∇**c. White dashed lines indicate channel boundaries. The inset shows the position of the focal plane (red dashed line) with respect to the PDMS microgrooved substrate. Scale bar = 100 μm. (b) Steady-state normalized fluorescence intensity profiles along the longitudinal direction. Red (blue) curves correspond to the presence (absence) of the steady salt concentration gradient, **∇**c. The location of the grooves with thickness *T* = 26 and pitch *L* = 65 is also shown.

Microgrooved silicon surfaces with high groove depth/thickness ratios (*H*/*T* > 2) are typically fabricated via deep reactive ion etching (DRIE),^50,51^ which is an expensive microfabrication technique. Conversely, PDMS substrates with high-aspect-ratio microstructures can be fabricated via soft-lithography replica molding,^52^ which is a faster and cheaper microfabrication process compared to DRIE. For this reason, we investigated whether diffusiophoresis-driven particle accumulation within grooves could be achieved also in PDMS devices. Particle manipulation experiments were conducted in PDMS microchannels fitted with microgrooves of thickness *T* = 26 μm and depth *H* = 45 μm (Table 1). The Ψ-junction channel geometry is identical to the one of the NOA-silicon devices. A colloid solution at low salt concentration (*c*_L_ = 0.1 mM) was used as the inner phase, whereas the outer phase was swapped between low (*c*_L_) and high (*c*_H_ = 10 mM) salt concentration solutions. Since our PDMS devices are relatively thick (>5 mm), it was not possible to determine the 3D particle distribution in the microchannels via confocal scanning, as this requires objectives with a high numerical aperture and low working distances (<1 mm). Therefore, the particle distribution in the PDMS device was assessed via epi-fluorescence microscopy. Figure 5a shows the epi-fluorescence micrographs of the PDMS microchannel at ca. 4 mm from the junction (*z*/*w* = 10) in the presence and absence of a salt concentration gradient, **∇**c. The focal plane was located at the entrance of the groove, as shown in the inset of the figure. Under this condition, the micrographs captured the steady-state fluorescence intensity of nanoparticles located either in the main channel or inside the microgrooves, at few microns from the groove entrance. Indeed, the depth of field of the microscope—that is, the thickness of the slice region that is in acceptably sharp focus in the micrographs—is given by^53^*d* = *n*_ind_λ_em_/NA^2^ + *n*_ind_*e*/(*M*·NA) ≃ 10 μm, where *n*_ind_ = 1 is the refractive index of the objective immersion medium (air), λ_em_ = 510 nm is the nanoparticle emission wavelength, *e* = 4.8 μm is the pixel pitch of the CMOS camera, and NA = 0.25 and M = 10 are the objective numerical aperture and magnification, respectively. In the absence of a salt concentration gradient, colloids are evenly distributed within the central region of the channel. The lower fluorescence intensity regions, corresponding to the grooves, suggest that the colloid transport within the grooves is hampered, likely due to steric and electrokinetic lift effects, as also observed for the silicon microgrooved surfaces. Conversely, in the presence of a salt concentration gradient, the colloids accumulate within the grooves and spread along the transverse direction. The phenomenon is fully reversible, and the original particle distribution can be recovered once the salt concentration gradient is removed. Figure 5b shows the normalized fluorescence intensity profiles along the flow (longitudinal) direction, *z*, without and with the salt contrast. The profiles were generated by averaging the micrograph intensity over the central region of the channel, – 0.2 < *x*/*W*_c_ < 0.2. Under iso-osmotic flow conditions (**∇**c = 0), the intensity profile has periodic oscillations with a wavelength matching the groove pitch, *L* = 65 μm. The intensity valleys overlap with the groove location, indicating a lower particle concentration within these regions. The same oscillatory trend with wavelength, *L*, is observed also for the profile under a steady salt concentration gradient, but the intensity valleys are replaced by peaks, demonstrating the accumulation of colloids within the microgrooves. Although epi-fluorescence imaging does not allow for the measurement of the groove trapping performance parameter, *A*, the latter can be roughly estimated as follows. The average fluorescence intensity of the micrographs at the groove locations can be approximated as *I̅*_groove_ ≃ 0.5(1 + *A*) (see the Supporting Information for derivation). According to Figure 5b, *I̅*_groove_ ≃ 1.6 ± 0.1) and, thus, *A* ≃ 2.2 ± 0.2, namely, the average particle concentration within the grooves is about twice the concentration of the original colloid solution injected in the device. This is a similar trapping performance to the one observed in shallower (*H* = 30 μm) silicon grooves.

To conclude, PDMS microgrooved channels can be successfully used for the steady and reversible accumulation of nanoparticles within dead-end grooves by diffusiophoresis. Compared with silicon substrates, PDMS microgrooved channels are a promising and advantageous alternative, due to their cheaper and easier microfabrication process, which can facilitate the development of new and cost-effective lab-on-chip technologies for colloidal particle transport, accumulation, and separation. To this end, design optimization studies will be needed to identify the optimal geometry of the PDMS microgrooved substrates, leading to the best performance for the examined application.

## Conclusions

Enhanced particle trapping in microgrooved channels was achieved via steady-state solute concentration gradients generated by continuous electrolyte flows past a microgrooved substrate. Steady-state particle distributions were observed within 2–3 min after switching the outer stream by means of a flow valve. Such a time lag is determined by the advective transport of the outer stream from the switching valve to the device. At the steady state, particle concentration peaks remain well below the packing limit, hence avoiding the risk of device clogging. The effects of groove depth and salt contrast on the particle accumulation process were assessed by measuring the distribution of nanoparticles in the channel and grooves via confocal laser scanning microscopy. Particle accumulation performance was quantified in terms of average particle concentration within the grooves and particle concentration peak intensity. Higher average particle concentrations and peak intensities were observed for more intense salt contrast and for grooves with lower thickness to depth ratios, *H*/*T*. The latter result is particularly advantageous since microstructures with high aspect ratios are more difficult and expensive to manufacture. Under enhanced particle trapping conditions, the average particle concentrations within the grooves were more than twice the bulk particle concentration in the flowing solutions and 75-fold higher than the average particle concentration within the grooves in the absence of a salt concentration gradients. Also, the particle concentration peak was more than four times the bulk value. Finally, we manufactured PDMS microgrooved channels with shallower grooves (depth to thickness ratio, *H*/T ≃ 2) via soft-lithography and showed effective diffusiophoresis-driven colloidal particle accumulation in these devices. PDMS substrates are much easier and cheaper to fabricate than silicon ones; thus, these promising systems can facilitate the translation of the proposed particle manipulation strategy into practical applications. Indeed, our microfluidic devices offer new opportunities for the exploitation of diffusiophoresis transport in soft matter and living systems for drug delivery, synthetic biology, and on-chip diagnostics applications. For instance, the proposed strategy could be applied for on-chip filtration and preconcentration of charged nanoparticles, such as polymer and metal nanobeads, micelles, DNA strands, liposomes, polymersomes, extracellular vesicles, and bacterial cells. Importantly, the reversibility of the trapping process would enable the rapid recovery of the filtered or preconcentrated sample, which could then be followed by off-chip downstream analysis (e.g., flow cytometry and SEM/TEM sample inspections).

## Availability of Data

The data that support the findings of this study are openly available on Loughborough University repository at http://doi.org/10.17028/rd.lboro.21513774.

## Appendix A

### Conditions for Particle Accumulation

Let us consider a steady one-dimensional configuration where particles are transported by the combined effect of diffusion, hydrodynamic advection, and diffusiophoresis. Regardless of the dimension considered, the divergence of the total flux, that is, the sum of the diffusion and advection fluxes—the latter including the diffusiophoresis flux—must be zero at any point in the domain

3

where *J*_D_ = −*D*_*n*_**∇***n* and *J*_c_ = (***u*** + ***u***_DP_) *n* are the diffusive and convective fluxes, respectively. In a 1D domain, this condition becomes

4

Integrating the previous equation yields

5

where the total flux of particles *J*_0_ (constant through the whole domain) is to be determined by boundary conditions. As shown by eq 5, the value of the constant *J*_0_ is determined by the values of the velocity, the particle concentration field, and its first derivative at the boundaries of the domain. At the location *x* = *x*_peak_ of the local maximum of the particle distribution, the first derivative of *n* with respect to the space coordinate must vanish—that is, d*n*/d*x* = 0. Therefore, according to eq 5 and assuming that the particle concentration is non-zero at the peak, it follows that the total velocity of particles at the peak is

6

Therefore, in a one-dimensional framework, the condition of zero particle total velocity *u* + *u*_DP_ = 0 determines the location of the particle concentration local extremum, only if *J*_0_ = 0 (which depends on the boundary conditions of the problem). Alternatively, when there is a steady flux of particles from one end of the domain to the other (i.e., *J*_0_ ≠ 0), the total particle velocity at the peak is not zero. Actually, the local extremum is located where condition eq 6 is satisfied.

To ensure that the local extremum at the location *x* = *x*_peak_ is a local maximum, the particle density must satisfy both conditions

7

From eq 5, it follows that, at *x* = *x*_peak_

8

since the divergence of the hydrodynamic velocity vanishes for an incompressible fluid (d*u*/d*x* = 0). By combining eqs 7 and 8, it follows that

9

We can thus draw the following conclusion. In a 1D steady-state configuration, with zero particle flux at the domain’s boundaries, the double condition

10

is both sufficient and necessary for having a local maximum in particle density.

Likewise, in the case of a non-zero total flux *J*_0_ and provided that the particle density *n* remains finite, the double condition

11

is both necessary and sufficient for having a local maximum in particle density.

## Supporting Information Available

Scanning electron microscopy images of PDMS microgrooved substrates; time evolution of the trapping performance parameter and particle concentration profile along the depthwise direction for three neighboring silicon grooves during two delivery/extraction cycles; and calculations of the particle accumulation rate and trapping performance via epi-fluorescence imaging (PDF)
