# Supplementary material for: Enhanced Accumulation of Colloidal Particles in Microgrooved Channels via Diffusiophoresis and Steady-State Electrolyte Flows
Source: Langmuir. 2022 Nov 9;38(46):14053–62. doi: 10.1021/acs.langmuir.2c01755 (PMC9686125; doi:10.1021/acs.langmuir.2c01755)
Supplement: Supplementary file 1 — la2c01755_si_001.pdf [file la2c01755_si_001.pdf]

Enhanced accumulation of colloidal particles in microgrooved channels via diffusiophoresis and steady-state electrolyte flows

Naval Singh,[†] Goran T. Vladisavljević,[†] François Nadal,[‡] Cécile Cottin-Bizonne,[¶] Christophe Pirat,[¶] and Guido Bolognesi[*,†]

†*Department of Chemical Engineering, Loughborough University, Loughborough, LE11 3TU, United Kingdom*

‡*Wolfson School of Mechanical, Electrical and Manufacturing Engineering, Loughborough University, Loughborough, LE11 3TU, United Kingdom*

¶*Institut Lumière Matière, UMR5306 Université Claude Bernard Lyon 1 - CNRS, Université de Lyon, Villeurbanne Cedex, 69622, France*

E-mail: g.bolognesi@lboro.ac.uk

Supplementary Information

PDMS microgrooved substrates

The PDMS microgrooved substrates were characterized by scanning electron microscopy. The substrates were sliced in half along the longitudinal axis to allow side-view imaging of the grooves. A typical tilted view of the microgrooved substrate is shown in Figure S1a. A side view of the substrate, shown in Figure S1, was used to measure the groove depth, thickness and pitch.

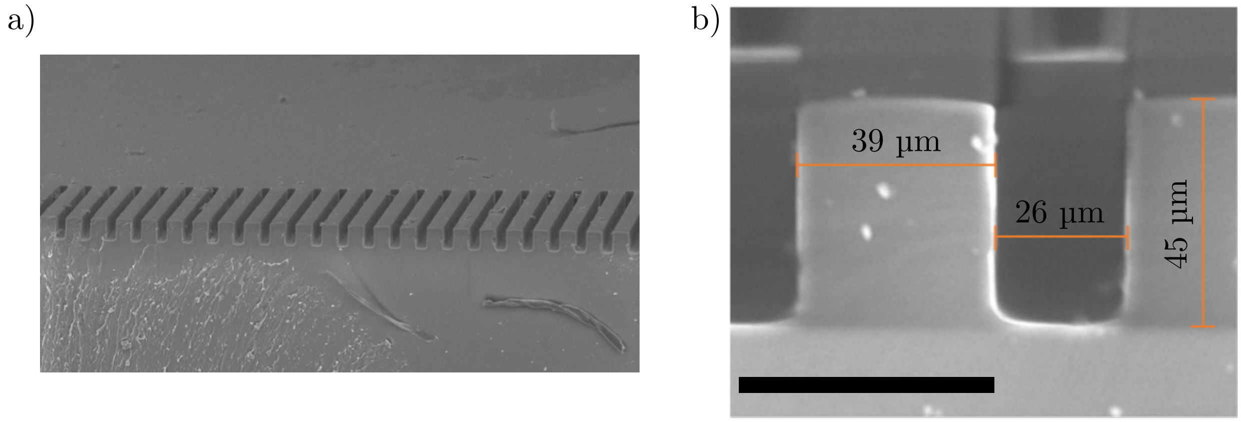

Figure S1: Scanning electron microscopy images of the PDMS microgrooved substrate. (a) Tilted view of a substrate sliced in half along the longitudinal axis. (b) Side view of a grooved substrate showing main dimensions. Scale bar = 50 µm.

Steady and reversible accumulation of colloidal particles

Figure S2a shows the time evolution of the trapping performance for three consecutive grooves when the side stream is swapped cyclically between low and high salt concentration solutions. The experiment was conducted with a NOA-silicon device with a channel depth, H_c = 57 µm, and a groove depth, H = 45 µm. The low and high salt concentrations were c_L = 0.1 mM and c_H = 10 mM, respectively. The time points at which the flow valve was switched to establish or remove the salt concentration gradient are highlighted in

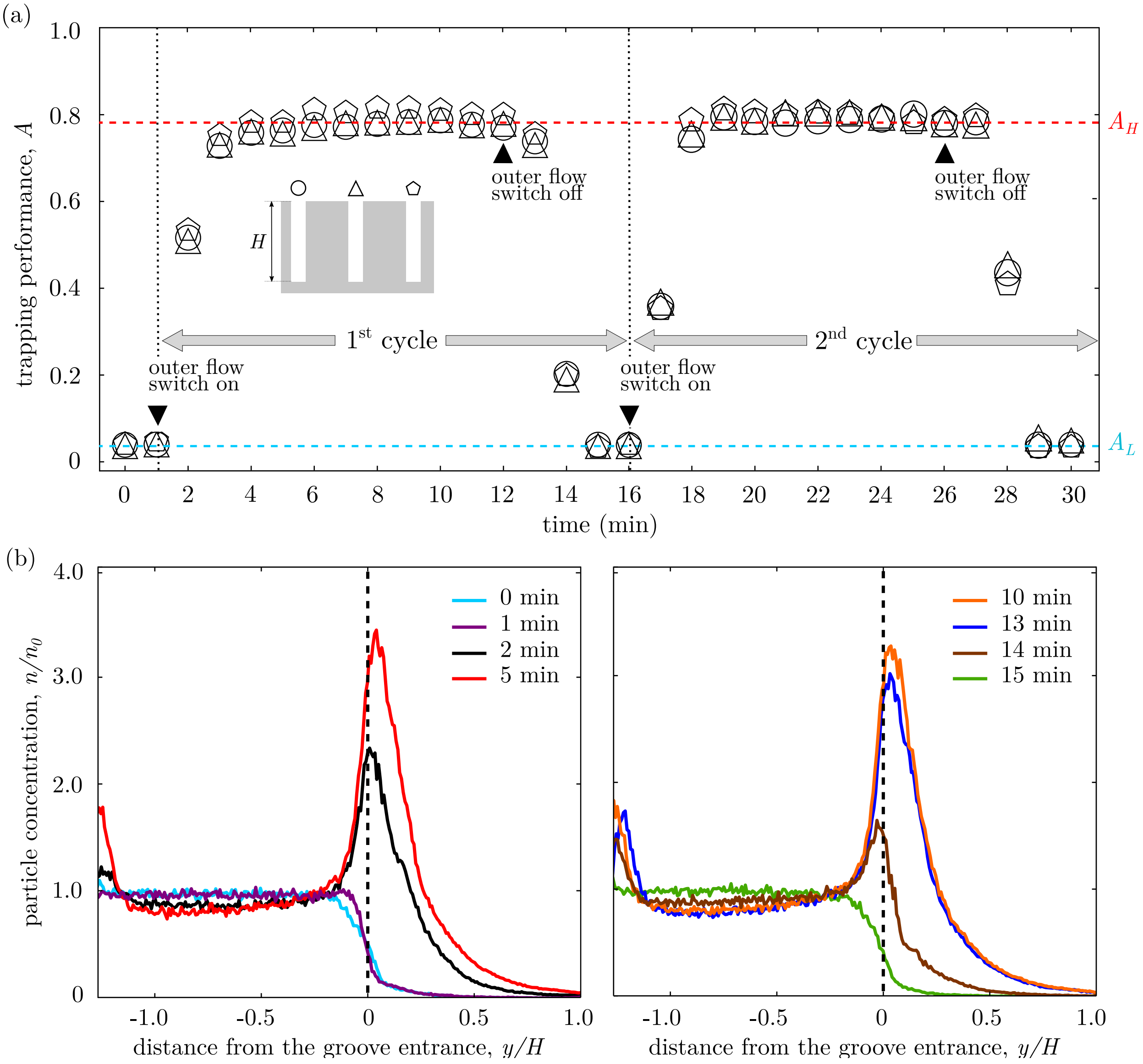

Figure S2: (a) Time evolution of the trapping performance of three neighbouring grooves (identified by different symbols) located at 4 mm from the junction, during two delivery/extraction cycles. The colloids are delivered and extracted from the groove by alternating the side fluid stream between high (c_H) and low (c_L) salt solutions. (b) Time evolution of the normalized particle concentration profile along the depth-wise direction for one groove, corresponding to the first delivery/extraction cycle. Channel depth, $H_c = 57$ μm; Groove depth, $H = 45$ μm ; Salt concentration in solutions, $c_L = 0.1$ mM and $c_H = 10$ mM.

the figure. The corresponding variation of the concentration profiles over time is shown in Fig. S2b for one of the grooves and for the first cycle only. A steady-state distribution of particles was achieved few minutes after the flow valve was switched, the trapping perfor-

mance plateauing at a value of $A_H = 0.79 \pm 0.03$. The trapping performance then dropped back to the initial value, $A_L = 0.022 \pm 0.002$, as the side stream was switched back to the low salt solution. Indeed, under iso-osmotic conditions, occurring at $t \in [12, 16]$ min and $t > 26$ min, the trapping mechanism ceased and the colloids could freely diffuse out of the microgrooves, until the trapping performance value reached A_L. We conclude that, as the flow configuration is cycled, the colloid concentration profile and the groove trapping performance change accordingly and return to the initial values at the end of each cycle. Hence, the particle accumulation is fully reversible, and the trapping and extraction of the colloidal particles in the microgrooves can be controlled by adjusting the salt contrast between the side and central streams.

Particle accumulation rate

At steady-state, the particle accumulation rate is zero, since particle advection is balanced by particle diffusion. On the other hand, the accumulation rate is non-zero during the transient regime of the accumulation process. To understand the mechanisms determining the accumulation rate in the transient regime, we estimated the characteristic time for particle accumulation within the grooves as follows. At first, it is assumed that the salt concentration field reaches the steady condition much faster than the particle concentration field, so that the salt concentration field can be considered stationary. This assumption is reasonable since LiCl salt diffusivity ($D_s = 1.35 \times 10^{-9}\,\mathrm{m^2/s}$) is much larger than the polystyrene nanoparticle diffusivity ($D_p = 2.25 \times 10^{-12}\,\mathrm{m^2/s}$). In the early stage of the accumulation process, particle advection is the dominant transport mechanism. As shown in Figure S3, the total advective flux of particles, which includes the hydrodynamic advection and the diffusiophoresis transport, can be estimated as $\dot{Q}_n \simeq n_0 W\, T\, (\boldsymbol{u} + \boldsymbol{u}_{\mathrm{DP}}) \cdot \mathbf{e}_y$, where $\boldsymbol{u} + \boldsymbol{u}_{\mathrm{DP}}$ is the total particle velocity, $\mathbf{e}_y$ the unity vector perpendicular to the groove entrance, and W and T the groove width and thickness, respectively (see Figure 1 of the manuscript). Neglecting the diffusive flux, one can estimate the characteristic time scale for particle accumulation

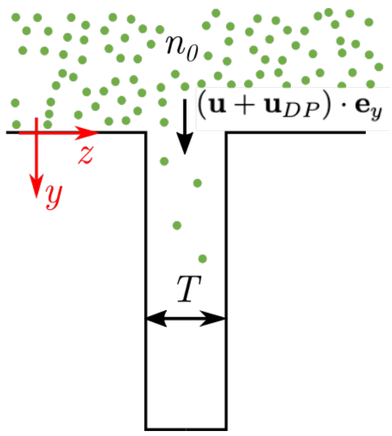

Figure S3: Particle advection from the microchannel to the groove at the early stages of the accumulation process.

as $\tau_{trans} \simeq \bar{n} V_G / \dot{Q}_n$, where $\bar{n} = n_0 A$ is the average particle concentration in the grooves, $V_G = H\,W\,T$ is the groove volume and H the groove depth. It follows

$$\tau_{trans} \simeq \frac{\bar{n}\,H\,W\,T}{n_0\,W\,T\,(\boldsymbol{u} + \boldsymbol{u}_{\mathrm{DP}}) \cdot \mathbf{e}_y} = \frac{A\,H}{(\boldsymbol{u} + \boldsymbol{u}_{\mathrm{DP}}) \cdot \mathbf{e}_y} \tag{S1}$$

According to our numerical simulations performed in a previous study,[1] the particle velocity component in the depth (y) direction, $(\boldsymbol{u} + \boldsymbol{u}_{\mathrm{DP}}) \cdot \mathbf{e}_y$, is ca. $5 \times 10^{-4}\,U_0$ with U_0 the average speed in the microchannel. Hence, $(\boldsymbol{u}+\boldsymbol{u}_{\mathrm{DP}}) \cdot \mathbf{e}_y \simeq 5 \times 10^{-4} U_0 \simeq 5 \times 10^{-4} \times 20\,\mathrm{mm/s} \simeq 10\,\mu\mathrm{m/s}$. For $A = 2.3$ and $H = 30\,\mu\mathrm{m}$, Eq. (S1) gives $\tau_{acc} \simeq 7\,\mathrm{s}$. This time scale is much smaller than transient time observed in the experiments (Figure 4), thereby suggesting that other mechanisms are controlling the particle dynamics in the unsteady state. To this end, let us account for the time required to deliver the higher salt concentration solution from the flow valve to the device (see also Figure 1 of the manuscript), once the valve is switched. The length and diameter of the tubing connecting the flow switching valve to the device inlet are $l_T \simeq 25\,\mathrm{cm}$ and $d_T = 0.01\,\mathrm{in} \simeq 250\,\mu\mathrm{m}$, respectively. The average speed in the tubing is $U_T = 4Q/\pi d^2 \simeq 4.2\,\mathrm{mm/s}$, where $Q = 12.5\,\mu\mathrm{L/min}$ is the flow rate of the outer stream. Therefore, it takes ca. $l_T/U_T \simeq 1\,\mathrm{min}$ for the higher salt concentration stream to reach the device after switching the flow valve. This time scale is of the same order of the

duration of the transient regime observed in the experiments (Figure 4). Once the higher salt concentration stream has reached the device inlet, the salt concentration field relaxes to steady state within few seconds ($H_c^2/D_s \simeq 2.4\,\mathrm{s}$ with $H_c = 57\,\mu\mathrm{m}$ the channel depth). Consequently, the particle accumulation rate is determined by the outer stream transport rate from the valve to the device rather than the particle advection and diffusiophoresis transport rate from the microchannel to the grooves.

Calculation of trapping performance via epi-fluorescence imaging

The evaluation of the trapping performance requires the measurement of the particle concentration profile along the groove depth-wise direction y, but the latter cannot be determined via epi-fluorescence imaging. To estimate the trapping performance from epi-fluorescence micrographs, one can approximate the particle concentration profile as follows

$$n(y)/n_0 = \begin{cases} 1 & \text{for} \quad y/H < 0 \\ A & \text{for} \quad y/H \geq 0 \end{cases} \tag{S2}$$

where $y = 0$ corresponds to the open end (i.e. entrance) of the grooves. The normalised intensity $I(x, z)$ of fluorescence micrographs with a focal plane located at the groove entrance ($y = 0$), can be written as

$$I = \int \mathcal{O}(y) n(y)/n_0 \mathrm{d}y \tag{S3}$$

where $\mathcal{O}(y) = \mathcal{O}_0 \cdot \left(\frac{\sin(u/4)}{u/4}\right)^2$ is the intensity distribution along the optical axis, with $u = \frac{2\pi}{\lambda}\mathrm{NA}^2 y$, λ the fluorescence emission peak, NA the numerical aperture of the objective. $\mathcal{O}_0$ is a normalising factor so that $\int \mathcal{O}(y)\mathrm{d}y = 1$. By replacing (S2) in (S3), it follows that the average fluorescence intensity at the grooves can be approximated as $\bar{I}_{groove} \simeq 0.5(1 + A)$.

References

(1) Singh, N.; Vladisavljević, G. T.; Nadal, F.; Cottin-Bizonne, C.; Pirat, C.; Bolognesi, G. Reversible trapping of colloids in microgrooved channels via diffusiophoresis under steady-state solute gradients. *Physical Review Letters* **2020**, *125*, 248002.
